# Ghosal Hematodiaphyseal Dysplasia: A Case Report

**Published:** 2020-04-01

**Authors:** Marjan Shakiba, Shahin Shamsian, Hamid Malekzadeh, Mehrdad Yasaei

**Affiliations:** 1 Department of Pediatric Endocrinology and Metabolism, Mofid Children’s Hospital, Shahid Beheshti University of Medical Sciences, Tehran, Iran; 2 Pediatric Congenital Hematologic Disorders Research Center, Mofid Children’s Hospital, Shahid Beheshti University of Medical Sciences, Tehran, Iran

**Keywords:** Ghosal syndrome, Anemia, Diaphyseal dysplasia

## Abstract

Ghosal hematodiaphyseal dysplasia (GHDD) is a rare autosomal recessive disorder presenting with steroid-responsive anemia and diaphyseal dysplasia of long bones. We report a 3-year-old Iranian girl with refractory anemia, splenomegaly and radiologic signs of metadiaphyseal dysplasia in long bones. The diagnosis was established by clinical presentation and X-ray bone survey. The patient was treated with oral prednisolone therapy with considerable improvement in anemia and splenomegaly.

## Introduction

 Ghosal hematodiaphyseal dysplasia syndrome is a rare variety of hypoplastic anemia. It was first described by ghosal et al. in five children with marked anemia and long bones thickening with radiographic features of metaphyseal and diaphyseal dysplasia^1^. GHDD is inherited by autsosmal recessive pattern with an ethnic background from Middle East and India^        2 ^. To date, more than 20 cases have been reported with this diagnosis and most of them were presented in the early childhood^   3 ^. Here, we report the clinical and radiologic findings in an Iranian child with GHDD and his response to treatment with prednisolone.

## Case presentation

A 3-year-old Iranian girl was referred to pediatric department due to progressive weakness, lassitude and pallor since the last 6 months. She was born at 38 weeks’ gestation to second degree consanguineous parents. At birth, her body weight was 2900 (10^th^-25^th^perentile) and head circumference was 35 cm (50^th^perentile). Past medical history was notable for chronic anemia diagnosed at 1 year of age without response to oral iron medication and other hematinics. There was no history of bleeding or bone pain. Her intellectual and motor development was normal. She was immunized due to national vaccination schedule with no history of complications.

On physical examination, she had pale skin and mucosa. Her weight and height were 12.5 kg (10^th^-25^th^ percentile) and 83 cm (<3^th^ percentile), respectively. There was no icterus, lymphadenopathy, and no purpuric spots were found on her skin. The spleen was palpable 6-7 cm under costal margin, but the liver span was within normal limits. There were no radial bones or thumb deformities. The physical examination was otherwise unremarkable.

Initial blood work showed a hemoglobin concentration of 5.7 g/dL, hematocrit of 16.5%, platelet count of 48000plt/µL, and leukocyte count of 4900 cells/µL (with a differential of 47% neutrophils, 1% bands, 45% lymphocytes and 6% monocytes). The reticulocyte count was 2% and the MCV was 76.8 fl. Liver function tests, uric acid, blood urea nitrogen, creatinine, and electrolytes levels were within normal limits. Peripheral blood smear showed normochromic, normocytic anemia with moderate thrombocytopenia. Iron studies and hemoglobin electrophoresis were normal. Results of direct Coombs test, G6PD, and osmotic fragility revealed no pathologic results. Serology tests for CMV, EBV and leishmaniasis were negative.

Bone marrow needle aspiration was obtained twice and both times revealed normocellularity with normoblastic erythropoiesis without atypical cells. Bone marrow biopsy showed unremarkable bone trabeculae with intervening severely hypocellular marrow.

**Figure 1:**
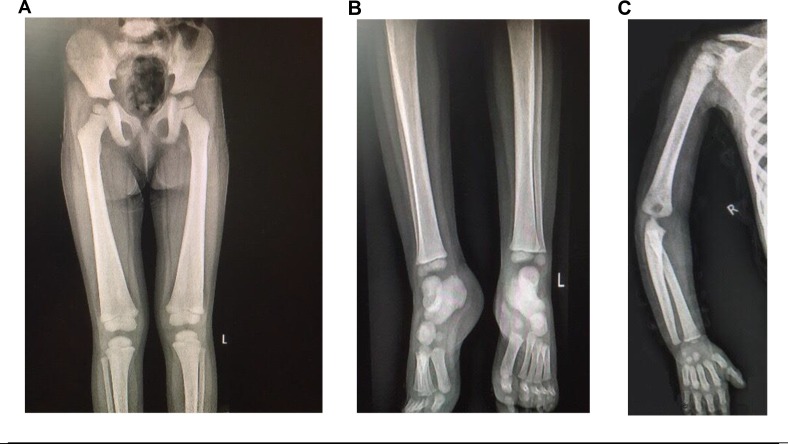
Bone survey. **A** and **B**:Radiographs of lower extermeties show femoral metadiaphyeseal widening in Erlenmeyer deformity pattern with endosteal thickenning of femurs and tibias.**C**:Diaphyseal widening of humerus and ulnrwith symetric increased cortical density.

Radiographic examination showed widening of diaphysis with symmetrical endosteal thickening of the long bones of extremities. Erlenmeyer deformity of distal femoral metadiaphysis was seen (Figure 1).

According to the diagnosis, prednisolone was initiated at a dose of 1 mg/kg/day (12.5 mg). With improvement in hemoglobin level in following visits, prednisolone was gradually tapered to 1.25 mg every other day (0.1 mg/kg/day) at her 4.5 year of age and continued as maintenance therapy. The response to corticosteroid was notable and after one year of treatment, hemoglobin level reached 15 g/dl. Moreover, the spleen was not palpable on physical examination.

## Discussion

 Ghosal hematodiaphyseal syndrome (GHDD; OMIM #231095) is a rare autosomal recessive disorder characterized by bone marrow dysfunction and increased long bone density with metadiaphyseal dysplasia ^2^^,^^4^ . Almost all of the GHDD cases reported to date have origins of the Indian subcontinent and Middle East with variable clinical features. The most consistent clinical presentations were steroid-responsive normochromic, normocytic anemia and diaphyseal dysplasia, mostly presented with endosteal thickening of long bones and widening of metadiaphysis. Other radiographic abnormalities such as Erlenmeyer flask-like deformity and base of skull sclerosis have also been reported ^4^^-^^6^ .

Bone marrow could be normocellularor hypocellular with some degrees of fibrosis, on which some authors have proposed GHDD as an etiology of myelophthisic anemia. Splenomegaly, detected in our patient, has been reported in some cases before and responded well to steroid therapy. Maintenance steroid therapy was needed in most of the cases, ranging from 0.1 mg/kg/day to 1.5 mg/kg/day  ^4^^, ^^5^ .

Research to discover the responsible gene of the disease revealed a homozygote mutation in the TBXAS1 gene on chromosome 7q33-34 causing impairment in the thromboxane-A synthase. As a participant in arachidonic acid metabolism pathway, defective thromboxane-A synthase leads to decreased thromboxane A2 (TXA2) level and subsequent increase in prostaglandin E2 level (PGE2). Elevated PGE2 may suppress erythroid precursor cells, resulting in refractory anemia seen in GHDD patients. It is also suggested that TXAS and TXA2 may also play a key role in bone remodeling as PGE2 that can stimulate both bone resorption and formation ^2^^,^^7^^,^^8^ .

Progressive diaphyseal dysplasia (PDD) also known as Camurati-Engelmann disease is a close radiologic differential diagnosis of GHDD. PDD is transmitted as an autosomal dominant trait and characterized by both endosteal and periosteal involvement and neuromuscular symptoms^   9 ^. In conclusion, GHDD should be in mind in the differential diagnoses of patients with anemia and skeletal dysplasia.
